# Supplementary material for: An evaluation of multi-excitation-wavelength standing-wave fluorescence microscopy (TartanSW) to improve sampling density in studies of the cell membrane and cytoskeleton
Source: Sci Rep. 2021 Feb 3;11:2903. doi: 10.1038/s41598-020-78282-6 (PMC7858599; doi:10.1038/s41598-020-78282-6)
Supplement: Supplementary file 1 [file 41598_2020_78282_MOESM1_ESM.pdf]

Supplementary Materials

An evaluation of multi-excitation-wavelength standing-wave fluorescence microscopy (TartanSW) to improve sampling density in studies of the cell membrane and cytoskeleton

Authors

Jana K. Schniete*[1], Peter W. Tinning[1], Ross C. Scrimgeour[1], Gillian Robb[1], Lisa S. Kölln[1], Katrina Wesencraft[1], Nikki R. Paul[2], Trevor J. Bushell[3], and Gail McConnell[1]

Affiliations

1 Department of Physics, SUPA, University of Strathclyde, 107 Rottenrow East, Glasgow, G4 0NG, UK

2 Cancer Research UK Beatson Institute, Garscube Estate, Switchback Road, Bearsden, Glasgow, G61 1BD, UK

3 Strathclyde Institute of Pharmacy and Biomedical Sciences, University of Strathclyde, 161 Cathedral Street, Glasgow, G4 0RE, UK

*corresponding author: jana.schniete@strath.ac.uk

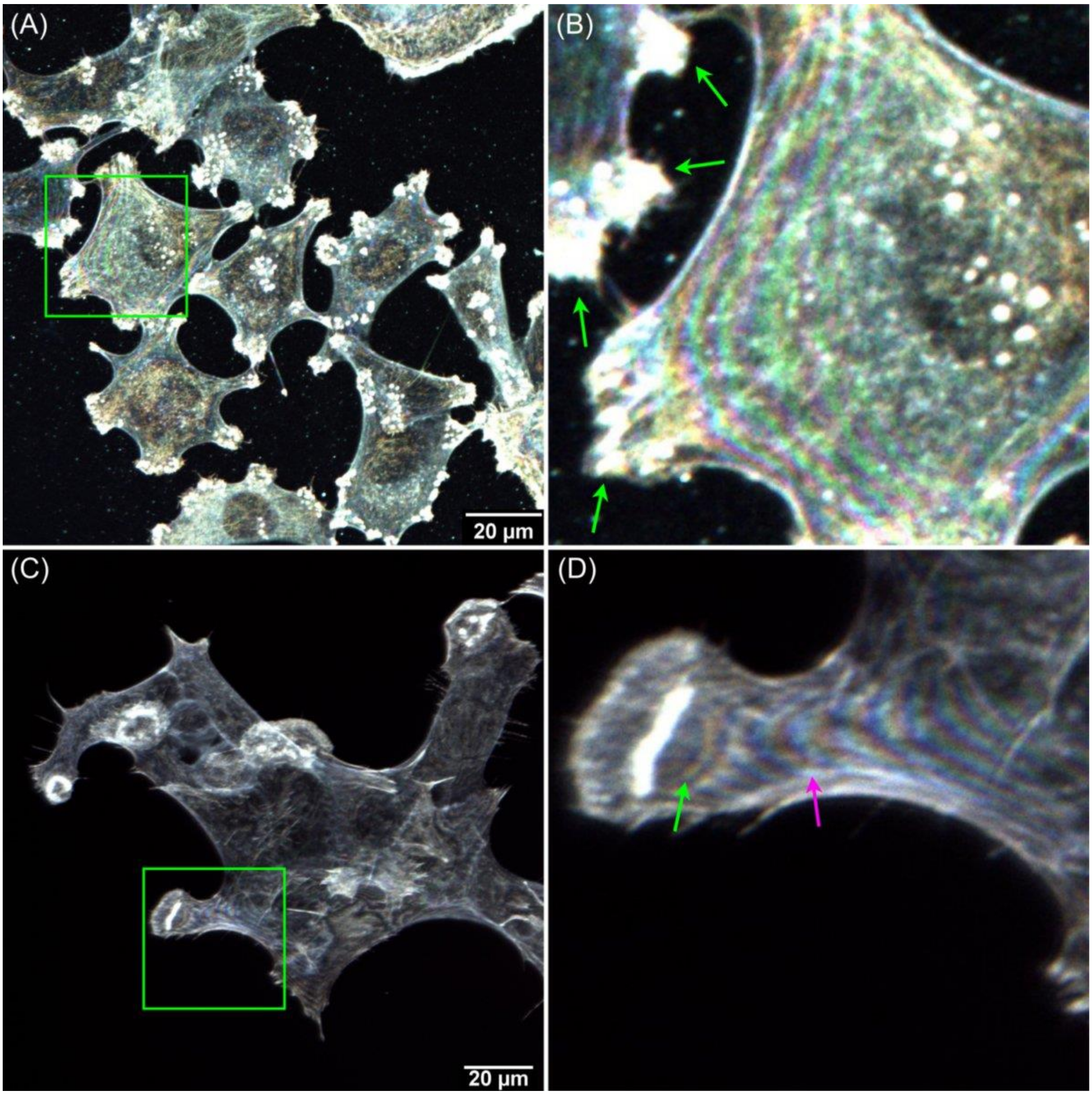

Supplementary Figure 1 Examples of TartanSW imaging of the actin network in fixed cell lines. (A) Pancreatic beta cell line 1.1B4 labelled with rhodamine-conjugated phalloidin. The 488 nm and 514 nm lines of an Argon laser and the 543 nm line of a Helium-Neon laser were used for excitation of fluorescence. Images were taken with 2048 x 2048 pixels, and were averaged over 8 frames at a line speed of 100 Hz, with an emission detection bandwidth of 550-650 nm. A green box indicates an ROI, which is cropped and magnified and shown in (B), where focal adhesions are indicated with green arrows. (C) Actin ruffles in fixed mouse PDAC (Pancreatic ductal adenocarcinoma) cells labelled with Alexa-488-conjugated phalloidin with a siRNA knockdown of Aldolase A (Aldoa). Fluorescence was excited with the 476 nm, 488nm and 496 nm lines from an Argon laser. Images were 4096 x 4096 pixels, and were averaged over 8 frames at a line speed of 100 Hz, with an emission detection bandwidth of 505-600 nm. A green box indicates an ROI, which is cropped and magnified and shown in (D). The magenta and green arrows highlight the ‘C’ and ‘D’ shaped curvature of two standing waves that indicate the presence of a tube-like structure beginning at the actin aggregate and proceeding into the cell body.

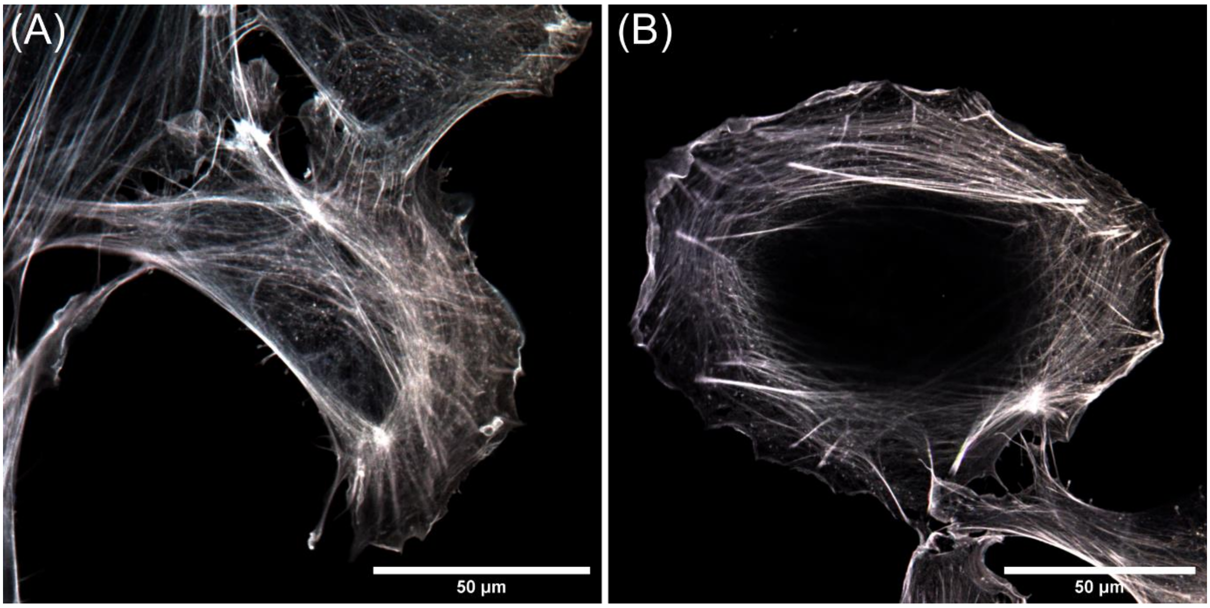

Supplementary Figure 2

Control studies confirm that the mirror is an essential part of the TartanSW method. Fixed 3T3 cells were grown on coverslips instead of a mirror, and were stained with rhodamine-conjugated phalloidin as control specimens. These preparations were imaged with the same parameters as described in Figure 3(E) and (F). (A) and (B) show two example RGB colour-merge images of cells. No pseudo-coloured anti-nodal planes are visible in the data.

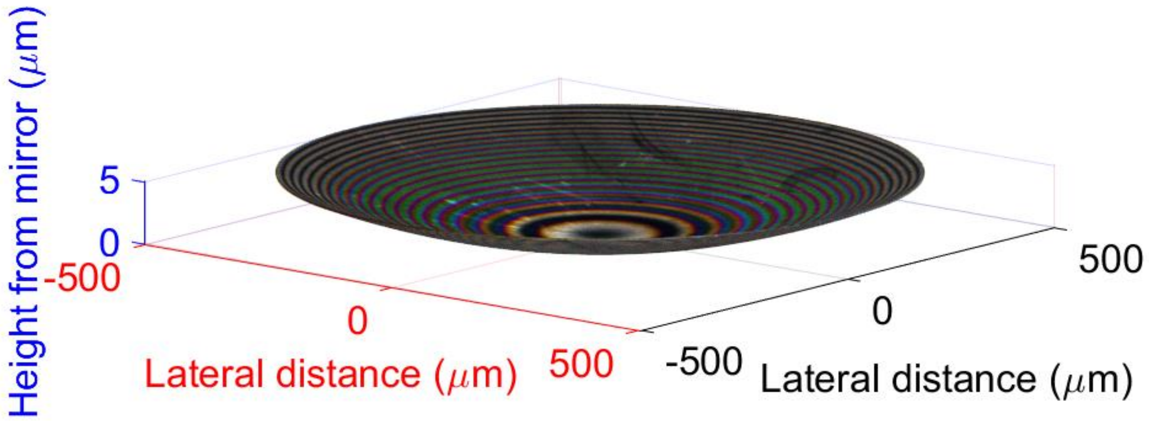

Supplementary Figure 3

3D reconstruction a lens specimen imaged using the TartanSW method from a 2D image using a custom written MATLAB code (see SF3) by generating two matrices containing the x and y positions of each pixel which where than converted to the corresponding height by a conversion factor. The radial distance, r, was then calculated from the x and y pixel distance matrices using Pythagoras theorem. The axial height of each pixel in micrometer was calculated from Eqn. 1 using the radial distance r, and the radius of the curvature, R, of the lens specimen. Lastly, a 3D reconstruction of the lens specimen was created using the x, y and z pixel values, and an RGB colour map obtained from the TartanSW image, using MATLABs scatter3 function

Supplementary videos - Live cell imaging using TartanSW

The videos available here: https://strathcloud.sharefile.eu/d-sbf0a4fbee364514b named as “Supplementary Video A SKOV-3 Lipilight_560.avi” and “Supplementary Video B 3T3 GFP_Lifeact.avi”

(A) Live SKOV-3 cells labelled with the membrane dye Lipilight 560. Images were obtained with 2048 x 2048 pixels over a period of 1 hour at 90 second intervals. A frame average of three was applied at a line speed of 100 Hz. The 488 nm and 514 nm lines of an Argon laser and the 543 nm line of a Helium-Neon laser were used for excitation of fluorescence, which was detected over an emission bandwidth of 550-650 nm.

(B) GFP LifeAct transfected 3T3 cells. Images were obtained with 1024 x 1024 pixels over a period of 40 minutes at 48 seconds intervals. A frame average of three was applied at a line speed of 100 Hz. The 476 nm, 488nm and 496 nm lines from an Argon laser were used for excitation and fluorescence was detected over an emission bandwidth of 520-625 nm.

Supplementary Files: Matlab Code

SF1 Matlab Code I: TartanLensanalysis.m

```
clc;
clearvars;

% Radius of Curvature of The lens specimen
R_lens = 0.02481;

% Calibration factor for the pixels per distance(micron)
calib = 1.39;
% Opens file explorer to load image file.
% Stores the image file directory.
% Reads in the image to the program.
[filename, pathname, filterindex] = uigetfile({'*.jpg;*.tif;*.png;*.gif;*.tiff','All Image Files'});
Filename = strcat(pathname,filename);
info = imfinfo(Filename);
imageStack = [];
numOfImages = length(info);

for k = 1:numOfImages
   currentImage = imread(Filename, k, 'Info', info);
   SWImage(:,:,k) = currentImage;
end

% Reads in the image size
[h,w,~] = size(SWImage);

% checks if the image is square.
if w==h
   z = SWImage;
elseif h > w
```

```
    h = w;
elseif w > h
    w = h;
end

% Checks if the width and height are even or odd. If they are odd
% one is minus from the width and height values
if mod(w,2)==1
    w2 = w-1;
else
    w2 = w;
end

if mod(h,2)==1
    h2 = h-1;
else
    h2 = h;
end

% Sets the new squared image size
z = SWImage(1:h2,1:w2,:);

for k = 1:3



    % sets the half width and half height values
    half_height = h2/2;
    half_width  = w2/2;
    m = half_height;

    % Create a radial average of the lens specimen
    [Zr(:,k), R(:,k)] = radialavg(z(:,:,k),m);
    radial_average(:,k) = (R(:,k)*half_height*1e-6)/calib;
```

```
    % Normalise the the fluorescence signal
    z_max(:,k) = max(max(Zr(:,k)));
    z_min(:,k) = min(min(Zr(:,k)));
    Zr_norm(:,k) = (Zr(:,k)- z_min(:,k))/(z_max(:,k)-z_min(:,k));

    % Translate the radial to axial height
    L(:,k) = (R_lens-sqrt(R_lens^2-radial_average(:,k).^2))*1e9;

    %% Analysis the FWHM and anti-nodal spacing
    % Finds the anti-nodal saa and FWHM of for each standing wave.
    [pks,locs,widths] = findpeaks(Zr_norm(:,k),L(:,k),'MinPeakProminence',0.05,'annotate','extents');

    %Determines the average FWHM of the standing wave planes
    Average_FWHM(:,k) = mean(widths);
    locs = locs';

    %Finds the size of the locs matrix
    [~,num_pks] = size(locs);

    %Calculates the anti-nodal-spacing for each peak
    Anti_nodal_spacing = (locs(1,2:num_pks)-locs(1,1:num_pks-1))';
    Avg_anti_nodal_spacing(:,k) = mean(Anti_nodal_spacing);

    clear pks locs x num_pks

end

% Plots Fluorescence Intensity vs Height from mirror
figure(2);plot(L(:,1),Zr_norm(:,1),'r')
hold on
plot(L(:,2),Zr_norm(:,2),'g')
hold on
plot(L(:,3),Zr_norm(:,3),'b')
hold off
```

```
% Axis labels
xlabel('Height from Mirror surface (nm)')
ylabel('Normalised fluorescence intensity (Arb. units)')
```

SF2 Matlab Code II– radialavg.m

```
function [Zr, R] = radialavg(z,m,xo,yo)
% RADIALAVG     Radially averaqe 2D square matrix z into m bins
%
% [Zr, R] = RADIALAVG(z,m,xo,yo)
%
% [Zr, R] = RADIALAVG(z,m,xo,yo) computes the average along the radius of a
% unit circle inscribed in the square matrix z. The average is computed in
% M bins. The radial average is not computed beyond the unit circle, in the
% corners of the matrix z. The radial average is returned in Zr and the
% mid-points of the M bins are returned in vector R. Not a Number (NaN)
% values are excluded from the calculation. If offset values xo,yo are
% used, the origin (0,0) of the unit circle about which the RADIALAVG is
% computed is offset by xo and yo relative to the origin of the unit square
% of the input z matrix.
%
% Example
%       N=101;
%       [X,Y] = meshgrid(-1:2/(N-1):1);
%       xo = +0.25;
%       yo = -0.25;
%       X = X-xo;
%       Y = Y-yo;
%       z = 1-sqrt(X.^2 + Y.^2);
%       m=(N-1)/2+1;
%       [Zr,R] = radialavg(z,m,xo,yo);
%       figure;plot(R,Zr,'.-');
%
```

```
% INPUT
% z = square input matrix to be radially averaged
% m = number of bins in which to compute radial average
% xo = offset of x-origin relative to unit square (DEF: 0)
% yo = offset of y-origin relative to unit square (DEF: 0)
%
% OUTPUT
% Zr = radial average of length m
% R  = m locations of Zr (i.e. midpoints of the m bins)
%
% See also linspace, meshgrid

% (c) 2014 David J. Fischer | fischer@shoutingman.com
% 4/4/14 DJF first working version
% 5/2/14 DJF documentation & radialavg_tester.m to demonstrate use
% radial distances r over grid of z
% 6/20/16 DJF Excludes NaN values
% 6/21/16 DJF Added origin offset

if ~exist('xo','var')
	xo = 0;
end
if ~exist('yo','var')
	yo = 0;
end

N = size(z,1);
[X,Y] = meshgrid(-1:2/(N-1):1);
X = X-xo;
Y = Y-yo;

r = sqrt(X.^2+Y.^2);

% equi-spaced points along radius which bound the bins to averaging radial values
```

```
% bins are set so 0 (zero) is the midpoint of the first bin and 1 is the last bin
dr = 1/(m-1);
rbins = linspace(-dr/2,1+dr/2,m+1);

% radial positions are midpoints of the bins
R =(rbins(1:end-1)+rbins(2:end))/2;

Zr = zeros(1,m); % vector for radial average
nans = ~isnan(z); % identify NaNs in input data

% loop over the bins, except the final (r=1) position
for j=1:m-1
        % find all matrix locations whose radial distance is in the jth bin
        bins = r>=rbins(j) & r<rbins(j+1);

        % exclude data that is NaN
        bins = logical(bins .* nans);

        % count the number of those locations
        n = sum(sum(bins));
        if n~=0
                % average the values at those binned locations
                Zr(j) = sum(z(bins))/n;
        else
                % special case for no bins (divide-by-zero)
                Zr(j) = NaN;
        end
end

% special case the last bin location to not average Z values for
% radial distances in the corners, beyond R=1
bins = r>=rbins(m) & r<=1;

% exclude data that is NaN
```

```
bins = logical(bins .* nans);

n = sum(sum(bins));
if n~=0
        % average the values at those binned locations
        Zr(m) = sum(z(bins))/n;
else
        Zr(m) = NaN;
End
```

SF3 Matlab Code III– 3Dreconstruction_lens.m

```
clc;
clearvars;

% Radius of Curvature of The lens specimen (meter)
R_lens = 0.02481;

% Calibration factor for the pixels per distance(px/um)
calib = 1.38;
% Opens file explorer to load image file.
% Stores the image file directory.
% Reads in the image to the program.
[filename, pathname, filterindex] = uigetfile({'*.jpg;*.tif;*.png;*.gif;*.tiff','All Image Files'});
Filename = strcat(pathname,filename);
info = imfinfo(Filename);
imageStack = [];
numOfImages = length(info);

if numOfImages == 1
   SWImage = mat2gray(imread(Filename));
else
   for k = 1:numOfImages
      currentImage = imread(Filename, k, 'Info', info);
```

```
        SWImage(:,:,k) = currentImage;
    end

SWImage = mat2gray(SWImage);

end

% Reads in the image size
[h,w,~] = size(SWImage);

% checks if the image is square.
if w==h
    z = SWImage;
elseif h > w
    h = w;
elseif w > h
    w = h;
end

% Checks if the width and height are even or odd. If they are odd
% one is minus from the width and height values
if mod(w,2)==1
    w2 = w-1;
else
    w2 = w;
end

if mod(h,2)==1
    h2 = h-1;
else
    h2 = h;
end

% define x and y coordinates around centre with zero at the image center
```

```
half_height = h2/2;
half_width = w2/2;

% this is done for symmetry
height = h2-1;
width = w2-1;

% create square RGB image
SWImage = SWImage(1:height, 1:width, :);

% create a x and y grid of pixel distances values from image centre
[x, y] = meshgrid((-half_width+1:half_width-1), (-half_height+1:half_height-1));

% translate the pixel distance to image scale and determine radial distance
x_dis = x/calib;
y_dis = y/calib;
r_dis = sqrt(x_dis.^2 + y_dis.^2).*1e-6;

% Calculate the theoretical height for each pixel based on the radius of
% curvature on the len specimen
z_dis = (R_lens-sqrt(R_lens^2-r_dis.^2)).*1e6;

% set remove any values of the reconstrution above the
% upper height boundary (This is done by pixel location than value thus it
% can be transfered to all matrixes
z_dis(z_dis > z_dis(1,half_height)) = NaN;
x_dis(z_dis > z_dis(1,half_height)) = NaN;
y_dis(z_dis > z_dis(1,half_height)) = NaN;
SWImage(z_dis > z_dis(1,half_height)) = NaN;

% Rearrange the x, y, z and colour from array RGB
x_array = x_dis(:);
y_array = y_dis(:);
z_array = z_dis(:);
```

```
% create colour map
R = SWImage(:, :, 1);
G = SWImage(:, :, 2);
B = SWImage(:, :, 3);

R_array = R(:);
G_array = G(:);
B_array = B(:);
c = cat(2, R_array, G_array, B_array);

%plot 3D reconstruction]
% figure('units','normalized','outerposition',[0 0 1 1])
H = scatter3(x_array,y_array,z_array,1,c,'.');
AX = gca();
AX.FontSize = 24;
set(AX,'XColor','r')
% set(AX,'YColor','g')
set(AX,'ZColor','b')
xlabel('Lateral distance (\mum)','FontSize',24,'Color','r')
ylabel('Lateral distance (\mum)','FontSize',24,'Color','g')
zlabel('Height from mirror (\mum)','FontSize',24,'Color','b')
view(40,12)
pbaspect([1 1 .1])
xlim([min(x_array) max(x_array)])
ylim([min(y_array) max(y_array)])
zlim([min(z_array) max(z_array)])
```
